# Pathogenic properties of human endometriotic epithelial cells require steroid receptor coactivator-2

**DOI:** 10.1093/reprod/xaag125

**Published:** 2026-10-06

**Authors:** Vineet K Maurya, Sivagami Gunasekaran, Nisha Misra, David M Lonard, Pooja Popli, Ramakrishna Kommagani, Samantha Hrbek, Song Yong, Niraj Joshi, Asgerally T Fazleabas, John P Lydon

**Affiliations:** Department of Molecular and Cellular Biology, Baylor College of Medicine, Houston, TX, United States; Department of Molecular and Cellular Biology, Baylor College of Medicine, Houston, TX, United States; Department of Molecular and Cellular Biology, Baylor College of Medicine, Houston, TX, United States; Department of Molecular and Cellular Biology, Baylor College of Medicine, Houston, TX, United States; Department of Pathology & Immunology, Baylor College of Medicine, Houston, TX, United States; Department of Pathology & Immunology, Baylor College of Medicine, Houston, TX, United States; Department of Obstetrics, Gynecology and Reproductive Biology, Michigan State University, Grand Rapids, MI, United States; Department of Obstetrics, Gynecology and Reproductive Biology, Michigan State University, Grand Rapids, MI, United States; Department of Obstetrics, Gynecology and Reproductive Biology, Michigan State University, Grand Rapids, MI, United States; Department of Obstetrics, Gynecology and Reproductive Biology, Michigan State University, Grand Rapids, MI, United States; Department of Molecular and Cellular Biology, Baylor College of Medicine, Houston, TX, United States

**Keywords:** endometriosis, steroid receptor coactivator-2, baboon, human, mouse, proliferation, migration

## Abstract

**In brief:** Although endometriosis is known to be a debilitating gynecological disease associated with chronic pelvic pain and infertility, our understanding of its molecular underpinnings is incomplete. Using experimentally induced nonhuman primate and murine endometriosis tissue along with a human ectopic endometriosis epithelial cell line, we reveal that steroid receptor coactivator-2 (and its downstream target S100A10) are important for the pathogenic properties of ectopic endometriotic epithelial cells, potentially providing new molecular vulnerabilities for future therapeutic invention.

**Abstract:** Commonly diagnosed in reproductive-age women, endometriosis is causally linked to subfertility, infertility, and chronic pelvic pain. Although theories exist on the etiopathogenesis of endometriosis, our understanding of its cellular and molecular underpinnings remain incomplete. We report that steroid receptor coactivator-2 (SRC-2), a member of the p160/SRC family of transcriptional coregulators, is expressed predominantly in epithelial cells of ectopic endometriotic lesions in the primate and mouse. Using an immortalized human endometriosis epithelial cell (iHEEC) line in conjunction with small interfering RNA–mediated silencing, we demonstrate that SRC-2 is essential for many of the pathogenic features ascribed to endometriotic lesion expansion, including sustained viability along with clonogenic, migratory, and invasive properties. Genome-scale iHEEC RNA-sequencing analysis implicates SRC-2 in a number of pathobiologies associated with the cellular phenotype of the ectopic endometriotic epithelial cell, including actin cytoskeleton reorganization. To underscore the utility of this RNA-sequencing dataset, we show that S100A10, a cytoarchitectural scaffolding protein, is responsive to SRC-2 and essential for many of the above cellular properties of the iHEEC that are ascribed to SRC-2. Because S100A10 dysregulation is strongly linked to the etiopathogenesis of a wide spectrum of malignancies, many of the molecular mechanisms that underlie S100A10’s role in cancer progression are predicted to be coopted in endometriosis. Therefore, these investigations highlight an unsuspected role for SRC-2 (and its transcriptome) in maintaining the pathogenetic phenotype of the ectopic endometriotic epithelial cell. Such findings support continued investigations of this coactivator and its downstream gene expression programs as potential targets for the prognosis, diagnosis, and/or treatment of this gynecological disorder in the future.

One of three members of the p160/steroid receptor coactivator (SRC) family (Leo & Chen, 2000; Szwarc et al., 2014b), SRC-2 (also designated NCOA2 and TIF2 in human; GRIP1 in mouse) is an evolutionary conserved primary transcriptional coregulator for a subset of nuclear and non-nuclear receptor transcription factors, which in turn govern distinct gene expression programs that underpin select tissue responses in vivo. Similar to SRC-1 and SRC-3, SRC-2 is organized into modular functional domains, the majority of which serve as interfaces for protein–protein interactions (Szwarc et al., 2014b). With this functional domain structure, SRC-2 primarily bridges distal enhancer-bound transcription factors to the basal transcriptional machinery at proximal promoter regions to enact RNA polymerase II–mediated transcription.

Early studies demonstrated that SRC-2 is expressed in the epithelium and stromal fibroblasts of the human endometrium (Gregory et al., 2002), indicating that SRC-2 exerts important cell type–specific roles in normal endometrial function. Subsequent functional investigations revealed that SRC-2 is critical for progesterone-dependent endometrial stromal cell decidualization (Kommagani et al., 2013; Szwarc et al., 2014a, 2018a), an indispensable cellular transformation process that is essential for the progression of the embryo implantation process and the establishment of the maternofetal interface (DeMayo & Lydon, 2020). Apart from stromal cell SRC-2, recent investigations show that epithelial cell SRC-2 is required for endometrial receptivity (Maurya et al., 2023), an early transient state that temporarily allows embryo attachment and adhesion to the apical surface of the epithelial lining of the endometrium. Together, these endometrial studies indicate that SRC-2 from the epithelial and stromal cell populations control distinct transcriptional responses, which together ensure successful embryo implantation.

As with other tissue pathologies—i.e., cancers of the prostate and breast (Cai et al., 2019; Qin et al., 2014; Xu et al., 2009), a subset of uterine tissue pathologies is associated with SRC-2. Early clinical investigations revealed that expression levels of SRC-2 (and SRC-3) are dysregulated in the epithelial and stromal compartments of endometrial tissue from a patient group predisposed not only to endometrial hyperplasia but also to infertility and subfertility (Gregory et al., 2002). Importantly, cell-based experiments have demonstrated that a number of human endometrial epithelial cancer cell lines require SRC-2 for optimum proliferation and anchorage-independent growth (Szwarc et al., 2018b) while mouse studies showed that perturbation of SRC-2 expression in the endometrium results in hyperplasia and a severe subfertility phenotype (Szwarc et al., 2014a). Collectively, these investigations provide compelling support for SRC-2 in the etiopathogenesis of a subset of endometrial pathologies linked with subfertility.

While not a malignancy, endometriosis presents a number of pathogenic properties similar to those of endometrial hyperplasia and cancer, including enhanced cell proliferation, migration, and invasion (Adilbayeva & Kunz, 2024; Bogani et al., 2025; Burghaus et al., 2015; Ellis & Wood, 2026, 2026; Klodnicka et al., 2025; Ochoa Bernal & Fazleabas, 2024). These shared pathogenic cellular functions prompted us to address whether SRC-2 is important for endometriotic cellular properties that are crucial for ectopic lesion expansion and invasion at distant anatomic sites.

We demonstrate that SRC-2 is essential for human endometriotic epithelial cell pathogenicity, encompassing sustained viability, migration, and invasion. Furthermore, genome-scale transcriptomics predict distinct gene expression programs that underpin SRC-2’s contribution to the pathogenetic features of human endometriotic epithelial cells, particularly actin cytoskeleton organization and extracellular matrix remodeling. To highlight the effectiveness of this transcriptome dataset, we found that S100A10 (or pII) is an SRC-2-responsive molecular target that is essential for many of the cellular pathogenic features ascribed to SRC-2 in the endometriotic epithelial cell. This finding is important since S100A10, a constitutively active member of the S100 calcium-binding protein family and indispensable for cytoskeletal homeostasis (Hedhli et al., 2012; Rescher & Gerke, 2008; Saiki & Horii, 2019), is now considered a promising early prognostic biomarker for a wide array of malignancies (Gao et al., 2022; Lokman et al., 2016; Ma et al., 2022; Noye et al., 2018; Tantyo et al., 2019).

## Materials and methods

### Baboon (Papio anubis) and mouse endometriotic tissues

Derived from a baboon model for endometriosis (*n* = 4), matched tissue samples from the eutopic uterus and ectopic endometriotic lesion (localized to pelvic peritoneal regions) were harvested. This nonhuman primate model for endometriosis involves autologous inoculation of menstrual-derived endometrial tissue into the peritoneal cavity (Fazleabas et al., 2004), modeling retrograde menstruation. For these investigations, both eutopic and ectopic tissues were obtained 15 months post–disease induction and during the midsecretory phase of the cycle (days: 9–12 postovulation) (D’Hooghe et al., 1994; Fazleabas, 2002, 2006). Significantly, the morphological and histological features of endometriotic lesions from the baboon endometriosis model closely parallel those reported for human endometriotic lesions (Fazleabas et al., 2002). All animal procedures used in these nonhuman primate studies were prospectively approved by the Institutional Animal Care and Use Committee (IACUC) at the University of Illinois, Chicago, and at Michigan State University (ACC #23-057).

Eutopic uterine tissue and ectopic endometriotic lesions were also investigated from a previously reported study using an established murine surgical model for endometriosis (Chadchan et al., 2019). For this rodent endometriosis model, uterine tissue from estrus-stage mice (*C57BL/6J* inbred strain) was autologously transplanted onto their peritoneal wall. After 3 weeks, the resulting endometriotic lesions appear as peritoneal cysts (Cummings & Metcalf, 1995; Korbel et al., 2010; Pelch et al., 2012), which resemble endometriotic lesions observed in human endometriosis (Fainaru et al., 2008; Korbel et al., 2010; Umezawa et al., 2009). Specifically, one uterine horn from 10-week-old estrus-stage mice (Taconic, Germantown, NY [*N* = 5]) was excised and longitudinally dissected. A dermal biopsy punch generated endometrial tissue fragments (3-mm diameter) that were sutured to the peritoneal wall of the same mouse (Fainaru et al., 2008; Kiani et al., 2018; Schreinemacher et al., 2012). Mouse surgeries were performed according to an animal protocol (# 20160227) approved by the IACUC at the Washington University School of Medicine.

### Immunohistochemical analysis

Prior to paraffin-embedding and sectioning onto glass slides, tissues were fixed overnight in 4% paraformaldehyde in phosphate-buffered saline (PBS). Immunohistochemical staining for SRC-2 and S100A10 was conducted using a primary rabbit anti-SRC-2 monoclonal antibody (#A300-345A; Bethyl Laboratories, Montgomery, TX; 1:500 dilution) and a mouse anti-S100A10 monoclonal antibody (#5529; Cell Signaling Technology Inc., Danvers, MA; 1:100 dilution), respectively. For detection of SRC-2 immunoreactivity in baboon and mouse tissues, the goat antirabbit secondary antibody was used (#P-1000; Vector Laboratories Inc., Burlingame, CA; 1:200 dilution). For visualization of S100A10 immunoreactivity in baboon tissue, a goat antimouse secondary antibody (#AI-9200; Vector Laboratories Inc.; 1:200 dilution) was employed. For mouse tissues, the M.O.M. (Mouse on Mouse) Immunodetection kit (#BMK-2202; Vector Laboratories, Inc.) was used for S100A10 immunodetection. As required, peroxidase activity was visualized using the Vectastain Elite ABC-horseradish peroxidase (HRP) kit (#PK-4000; Vector Laboratories Inc.). Following immunohistochemical staining, tissue sections were lightly counterstained with hematoxylin prior to the application of permount mounting medium and subsequent affixing of coverslips. For counting immunostained cells, a total of 300 cells from four different histological subfields (200× magnification) per baboon eutopic and ectopic tissue (*n* = 4 per group) were counted by two separate investigators, both blind to the tissue identities. The cell counting above was repeated in triplicate before average count values from each biological replicate were presented as a histogram.

### Immortalized human endometriotic epithelial cell line

The immortalized human endometriotic epithelial cell line (iHEEC/Luc; referred to as iHEEC hereafter) has been described previously (Bono et al., 2012; Maurya et al., 2022). The iHEEC line was generated from a human patient–derived ovarian endometrioma and immortalized using the human telomerase reverse transcriptase (*hTERT*) gene (Bono et al., 2012). For these experiments, the iHEEC line was cultured in Dulbecco’s modified Eagle medium/F12 medium supplemented with 10% fetal bovine serum (FBS; # F2442; Sigma-Aldrich, St Louis, MO) and a 1% penicillin–streptomycin antibiotic cocktail (#15140122; ThermoFisher Scientific Inc., Waltham, MA); medium was changed every 48 hr. Through use of the American Type Culture Collection cell line authentication service, the iHEEC line was verified by short tandem repeat profiling analysis.

### Immunocytochemistry

Cells were grown on coverslips and fixed with 4% paraformaldehyde in PBS for 20 min. Fixed cells were washed three times with PBS before permeabilization with 0.1% Triton X-100 in PBS for 15 min. Cells were then washed in PBS and blocked using a BOXALL solution (#SP-6000; Vector Laboratories Inc., Burlingame, CA) for SRC-2 or S100A10 staining. After blocking and subsequent washing, a rabbit polyclonal anti-SRC-2 primary antibody (#A300-345A; Bethyl Laboratories, Montgomery, TX; 1:500 dilution) was used. For S100A10 immunostaining, a mouse S100A10 monoclonal antibody (#5529; Cell Signaling Technology Inc., Danvers, MA; 1:200 dilution) was used. For SRC-2 immunodetection, an antirabbit secondary antibody was applied (#P-1000; Vector Laboratories Inc.; 1:200 dilution), and the peroxidase activity was visualized using the Vectastain Elite ABC-HRP kit (#PK-400; Vector Laboratories Inc.). For S100A10 immunodetection, a goat antimouse secondary antibody (#AI-9200; Vector Laboratories; 1:400 dilution) was used; the peroxidase activity was visualized using the Vectastain Elite ABC-HRP kit as described above. Following immunohistochemical staining, cells were lightly counterstained with hematoxylin prior to the application of permount mounting medium and subsequent coverslip overlay.

### Small interfering RNA transfections

In triplicate, iHEECs were cultured in six-well plates prior to transfection with 60 pmol of the nontargeting (*NT*) small interfering RNA (siRNA) (#D-001810-10-05; Dharmacon Inc., Lafayette, CO) or siRNAs targeting either human *SRC-2* (L-020159-00-0005) or *S100A10* (L2-0011766-01-005) (both Dharmacon) using the Lipofectamine RNAiMax transfection reagent (#13778100; Invitrogen Corporation, Carlsbad, CA) (Maurya et al., 2022). Forty-eight hours after siRNA transfection, cells were collected and processed for either quantitative real-time (qRT)-PCR, RNA-sequencing (RNA-seq), or western immunoblot analysis. Cells were also trypsinized and replated for cytological immunostaining as well as to assay for cell viability, clonogenic, migration, or invasion capabilities (Maurya et al., 2022).

### Quantitative real-time PCR

Human endometriotic epithelial cells were processed for total RNA preparation using the Purelink RNA Mini Kit (#12183020; ThermoFisher Scientific Inc.). For total RNA quantification, the Nano-Drop 2000 UV/Visual spectrophotometer (ThermoFisher Scientific Inc.) was used before RNA (1 µg) was reverse transcribed using the High-Capacity cDNA Reverse Transcription Kit (#4368814; ThermoFisher Scientific Inc.). The concentration of amplified cDNA was adjusted to 10 ng/µL before qRT-PCR was conducted using the Fast TaqMan 2X Mastermix kit (#4444557; Applied Biosystems/Life Technologies, Grand Island, NY). All qRT-PCR experiments were performed using the 7500 Fast Real-time PCR system (Applied Biosystems/Life Technologies); the delta-delta cycle threshold was used to normalize expression to the GAPDH reference.

### RNA expression analysis

Genome-scale RNA-seq and analysis were conducted as reported previously (Maurya et al., 2022). In summary, total RNA purity and physical integrity were assayed using the NanoDrop spectrophotometer (ThermoFisher Scientific Inc.) and the 2100 Bioanalyzer with RNA chips (Agilent Technologies, Santa Clara, CA), respectively. In these experiments, RNA samples scoring a RNA integrity number of ≥8 were included. For each experimental group, RNA samples from three replicates were used. Sequencing libraries were generated with the TruSeq Stranded mRNA kit (Illumina Inc., San Diego, CA) from 250 ng of RNA and then PCR amplified. Quality analysis of resultant libraries was executed using the 4200 TapeStation with D1000 ScreenTape assays (Ilumina Inc.). Adapter-ligated fragment concentration was determined by qRT-PCR assay with the KAPA Library Quantification Kit (KAPA Biosystems, Wilmington, MA). After equimolar pooling, libraries were quantified on the 2100 Bioanalyzer (using the High Sensitivity DNA kit and DNA chips) and the KAPA Library Quantification kit. Sequencing of libraries was performed on the NovaSeq platform (Illumina Inc.). Raw sequenced reads in Illumina fastq file format were aligned to the human genome (Genome Reference Consortium Human Build hg38; National Center for Biotechnology Information [NCBI]) using the universal RNA-seq aligner HISAT2 (Kim et al., 2019). The Python-based software package HTSeq was used to determine the number of reads that aligned to known genes through use of the ultrafast universal RNA-seq aligner: spliced transcripts alignment to a reference (Anders et al., 2015; Dobin et al., 2013 (http://www-huber.embl.de/users/anders/HTSeq). To reduce possible PCR bias, read duplicates were removed with Picard Tools (http://broad.institute.github.10/picard).

The Bioconductor package EdgeR was applied to the gene expression data to detect differentially expressed genes (DEGs) between groups (Robinson et al., 2010). The false-discovery rate (FDR) of DEGs was estimated using the Benjamini and Hochberg method (Benjamini & Hochberg, 1995). Gene expression comparisons with an FDR ≤ 0.05 and an absolute fold-change (IFCI) ≥ 1.2 were considered to be significantly differentially expressed between the two groups. Genes with significantly altered expression (IFCI ≥ 1.2; FDR ≤ 0.05) between the two groups were used to identify affected pathways (Huang da et al., 2009). Fragments per kilobase of transcript per million values of transcripts were used for hierarchical clustering; the pheatmap package in R was used to generate the clustergram. Using raw gene count data, principal component analysis was performed with the R function prcomp package (https://cran.r-project.org). All raw data files were deposited in Gene Expression Omnibus repository at the NCBI (GSE: 334431; www.ncbi.nlm.gov/geo). Gene Ontology enrichment analysis was performed using the DAVID (Database for Annotation, Visualization, and Integrated Discovery) functional annotation clustering tool (http://david.abcc.ncifcrff.gov) (Sherman et al., 2007). Biological pathways involving genes that are overexpressed in datasets of DEGs were identified using the Kyoto Encyclopedia of Genes and Genomes (KEGG; https://www.genome.jp/kegg/) (Kanehisa et al., 2016). The KEGG Orthology-Based Annotation system (KOBAS 2.0) was used (Xie et al., 2011).

### Immunoblot analysis

Twenty micrograms of cell lysate protein was separated on 4%–15% sodium dodecyl sulfate-polyacrylamide gels prior to transfer to polyvinylidene membranes. After transfer, polyvinylidene membranes were incubated for 1 hr in 5% nonfat dry milk (#sc-2324 [Blotto]; Santa Cruz Biotechnology Inc., Dallas, TX) in Tris-buffered saline with Tween 20 (TBS-T). Membranes were then incubated overnight at 4 °C with either one of the following rabbit or mouse primary antibodies: anti-SRC-2 antibody (#96687) or anti-S100A10 antibody (#5529; Cell Signaling Inc., Danvers, MA) each diluted 1:1,000 and anti-GAPDH (#MA5-1538; ThermoFisher Scientific Inc.; 1:10,000 dilution) in 5% nonfat milk in TBS-T. Membranes were then incubated with an antirabbit IgG secondary antibody (#A27036; ThermoFisher Scientific Inc.; 1:5,000 dilution) in 5% nonfat milk in TBS-T for 1 hr at room temperature. Chemiluminescence was visualized using the SuperSignal West Pico PLUS Chemiluminescent Substrate kit (#1863097; ThermoFisher Scientific, Inc.). Bands of specific immunoreactivity were digitally captured using the Bio-Rad ChemiDoc imaging system (Bio-Rad Laboratories, Hercules, CA).

### The MTT assay

At a density of 5 × 10^3^ cells per well, cells were seeded in 96-well culture plates in triplicate. Cells that were transfected with siRNAs for 48 hr were further cultured for 0, 24, 48, 72, or 96 hr before cell proliferation was measured using the CellTiter 96® Non-Radioactive Cell Proliferation Assay kit (#G4000; Promega Inc., Madison, WI). For each time point in culture, 15 µL of 3-(4,5-dimethylthiazol-2-yl)-2,5-diphenyltetrazolium bromide (MTT) (# M6494; Promega, Madison, WI) was added to each well for a final concentration of 0.5 mg/mL; MTT-treated cells were left for an additional 3 hr in the dark at 37 °C. The supernatant was subsequently removed and formazan crystals in each well were dissolved with the addition of the stop/solubilizing solution (100 µL (dimethyl sulfoxide/well), an additional 15-min incubation period at 37 °C with gentle agitation was conducted. Using a 96 well microtiter plate enzyme-linked immunosorbent assay reader, the absorbance was recorded at 570 nm (the formazan absorbance maximum wavelength) (Campling et al., 1988). The mean absorbance at “N” time point/mean absorbance at 0 hr: N = 0, 24, 48, 72, and 96 hr was used to calculate the relative cell proliferation. Each experiment was repeated at least three times with three to five technical replicates per treatment group.

### Colony-forming assay

Following siRNA transfection, cells were cultured in six-well plates (3 × 10^3^ cells per well in triplicate). After a 10-day culture period, cells were fixed with 4% paraformaldehyde for 15 min. Fixed cells were washed with PBS for 10 min prior to colonies being stained with a crystal violet solution (0.5%) for 15 min (Maurya et al., 2022). After a wash step, stained colonies were imaged. Crystal violet was eluted using 30% acetic acid; the eluent was transferred to a 96-well plate and absorbance read at 590 nm. Each experiment was repeated three times with triplicates for each treatment group.

### Transwell cell invasion assay

The Corning BioCoat Matrigel Invasion Chamber kit (#354480; ThermoFisher Scientific Inc.) was used to analyze cell invasion. After siRNA transfection, cells were resuspended in Opti-MEM medium. Culture medium with 20% FBS was added (0.6 mL) to the bottom of each transwell of the invasion chamber plate. The resuspended cells (1 × 10^5^ cells/250 µL) were then added onto each transwell insert surface in the upper chamber and allowed to invade. Following 48 hr of incubation at 37 °C, cells retained on the upper surface of the transwell insert (facing the upper chamber) were removed using a cotton swab. Invasive cells located on the lower surface of the transwell insert (facing the lower chamber) were fixed in 4% paraformaldehyde in PBS for 15 min before staining with a crystal violet solution for 10 min (Justus et al., 2014). Following washing with distilled water, transwell inserts were imaged using a Zeiss Stemi stereomicroscope with an attached AxioCam 305 color digital camera and ZEN software (Zeiss, Jena, Germany). Bound crystal violet was eluted by adding 300 µL of 30% acetic acid onto each insert and shaking for 10 min. The eluent was transferred to a 96-well clear microplate, and the absorbance read at 590 nm. Each experiment was repeated three times with triplicates for each group (Pijuan et al., 2019).

### Transwell cell migration assay

Cell migration was assessed using the Corning Transwell Chamber (#3422; Corning Inc., Glendale, AZ). Cells were seeded in six-well culture plates and cultured to 70%–80% confluency before siRNA transfection. Forty-eight hours post-transfection, cells were trypsinized and resuspended in culture medium. Cells were then counted and diluted to a density of 1 × 10^5^ cells/250 µL in Opti-MEM medium. Subsequently, 250 µL of the cell suspension was seeded into the upper chamber of each transwell. The lower chamber contained 600 µL culture medium with 20% FBS, which served as a chemoattractant. Cells were cultured in a humidified incubator at 37 °C with 5% CO_2_ for 24 hr. Transwell inserts were then washed twice with PBS before cells on the upper surface of the transwell insert (facing the upper chamber) were gently removed using a moistened cotton swab. Cells on the lower surface of the transwell insert (facing the lower chamber) were fixed in 4% paraformaldehyde in PBS for 15 min prior to staining with crystal violet; migrated cells were digitally imaged. Bound crystal violet was then eluted by adding 300 µL of 30% acetic acid onto each transwell insert and shaking for 10 min. The eluent was transferred to a 96-well microplate and absorbance measured at 590 nm using a plate reader. Experiments were conducted three times with triplicate samples for each treatment group.

### Statistical analysis

To calculate the statistical significance of differences between two groups, two-tailed unpaired Student *t*-tests were used. For the majority of experiments, data were graphically displayed as the mean ± standard deviation. Differences between groups with *p-*values <0.05 were considered statistically significant; asterisks indicate the level of significance: *p < 0.05, **p < 0.01, ***p < 0.001, and ****p < 0.0001. The Prism software package (version 9) (GraphPad Software Inc., San Diego, CA) was used for most of the statistical analyses reported herein.

## Results

### Epithelial cells of baboon and murine endometriotic lesions express SRC-2

Immunohistochemical analysis clearly reveals that the majority of SRC-2 immunoreactivity is localized to the epithelial compartment of experimentally induced endometriotic lesions from both the baboon (Figure 1) and the mouse (Figure S1). In the case of the eutopic endometrium, SRC-2 is expressed in both the epithelial and stromal cell compartments; however, the intensity of the immunostaining for SRC-2 in the stromal compartment is markedly lower when compared with the epithelial compartment. These descriptive results suggest that the epithelial SRC-2 population may contribute to the pathogenic functions of endometriotic lesions. To test this proposal, the pathogenic properties of an iHEEC line (Bono et al., 2012) were investigated, in which *SRC-2* levels were significantly decreased through siRNA-mediated silencing.

**Figure 1:**
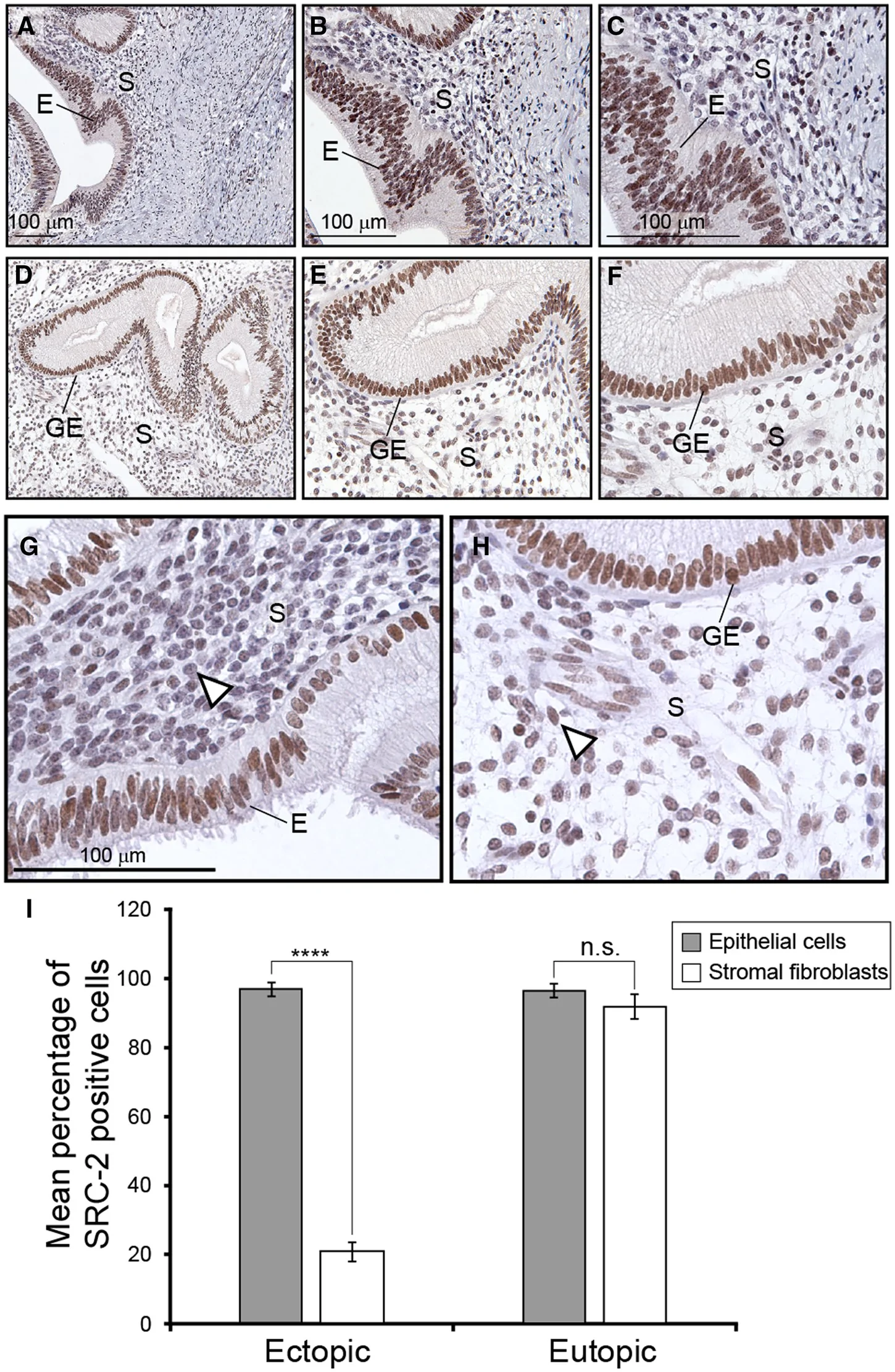
Epithelial cells are the predominant cell type in the baboon ectopic lesion that expresses steroid receptor coactivator-2 (SRC-2). (A) Low-power magnification shows that the majority of SRC-2 expression is localized to the epithelial cell layer of the endometriotic lesion; E and S denote epithelium and stroma, respectively. (B) Higher magnification of a subfield in the image shown in panel (A); note that the majority of SRC-2 immunopositivity is localized to the epithelial layer. (C) Higher power magnification of a section the epithelial layer shows obvious SRC-2 immunoreactivity in the epithelium (E) with low SRC-2 expression within the underlying stromal (S) cell layer. (D–F) Increasing high-power magnification images of SRC-2 immunohistochemical staining of matched eutopic endometrial tissue. Note that a subset of glandular epithelial (GE) cells score positive for SRC-2 expression with lower but detectable SRC-2 expression in the stroma (S). Scale bar in panels (A–C) apply to panels (D–F), respectively. (G) High-power magnification of a region within the ectopic lesion that shows strong SRC-2 expression in the epithelium with comparatively negligible SRC-2 staining in the underlying stroma (white triangle). (H) High-power magnification of a subfield in the eutopic endometrium that shows clear SRC-2 immunoreactivity in the GE with lower SRC-2 immunostaining in the stromal compartment (white triangle); scale bar in (G) applies to (H). The immunohistochemical results represent one replicate from four replicates analyzed. (I) The histogram displays the mean number of epithelial and stromal cells that are immunopositive for SRC-2 expression in eutopic and ectopic endometriotic tissues obtained from four individual baboons. Cell counting was performed on four different subfields within each histological section and expressed as the mean percentage positive for SRC-2 expression.

### Pathogenic functions of iHEECs require SRC-2

Immunohistochemistry confirmed that SRC-2 protein is expressed in the majority of iHEECs, and that siRNA-mediated knockdown significantly reduced *SRC-2* levels in this cell type (Figure 2A). Quantitative real-time PCR confirmed that the reduction in SRC-2 protein levels was due to decreased levels of *SRC-2* transcripts following the 48 hr *SRC-2* siRNA transfection period (Figure 2B). Importantly, *SRC-2* knockdown did not alter the expression levels of *SRC-1* or *SRC-3* (Figure S2). Based on these findings, we next investigated whether SRC-2 is necessary for the sustained viability and colony-forming abilities of iHEECs. The MTT and colony formation assays demonstrated that SRC-2 is required for sustained viability of iHEECs over a prolonged culture period and for the clonogenic abilities of this cell type respectively (Figure 2C–E). Transwell migration and invasion assays demonstrated the importance of SRC-2 for the migratory and invasive properties of iHEECs (Figure 3). Together, these results support an indispensable role for SRC-2 in many of the pathogenic cellular behaviors ascribed to the endometriotic epithelial cell.

**Figure 2:**
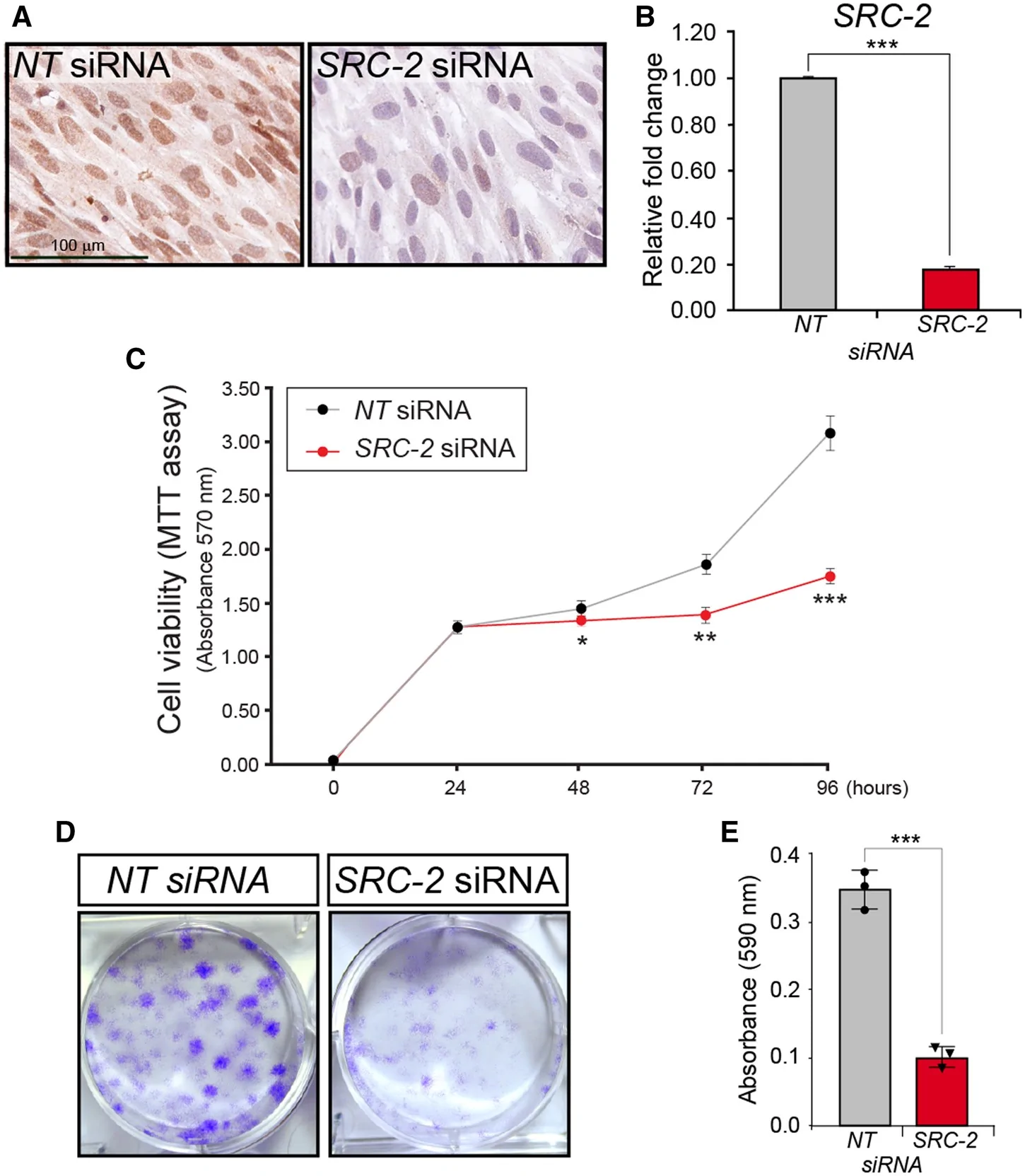
Cell viability and colony-formation properties of immortalized human endometriosis epithelial cells (iHEECs) are significantly compromised when steroid receptor coactivator-2 (SRC-2) levels are reduced. (A) Left panel shows a typical immunocytological staining result that reveals significant SRC-2 protein expression in the majority of iHEECs following nontargeting (*NT*) small interfering RNA (siRNA) transfection. Right panel shows that iHEECs exhibit a marked decrease in SRC-2 protein expression following *SRC-2* knockdown. (B) Representative quantitative PCR results following *NT* or *SRC-2* knockdown in iHEECs for 48 hr. Note that *SRC-2* RNA levels are markedly reduced with *SRC-2* knockdown. (C) Viability of iHEECs was assessed by the MTT (3-[4,5-dimethylthiazol-2-yl]-2,5-diphenyltetrazolium bromide) assay; note the significant reduction in cell viability with *SRC-2* knockdown. (D) A typical result from a colony-formation assay using the iHEEC line that was transfected with *NT* or *SRC-2* siRNAs for 48 hr, followed by a 10-day culture period. (E) Histogram shows the mean absorbance (590 nm) of the crystal violet eluent from stained iHEEC colonies following *NT* or *SRC-2* knockdown. For the above histograms, results are displayed as the mean ± standard deviation and are representative of three separate experiments; *p-value <0.01; **p-value <0.001; and ***p-value <0.0001.

**Figure 3:**
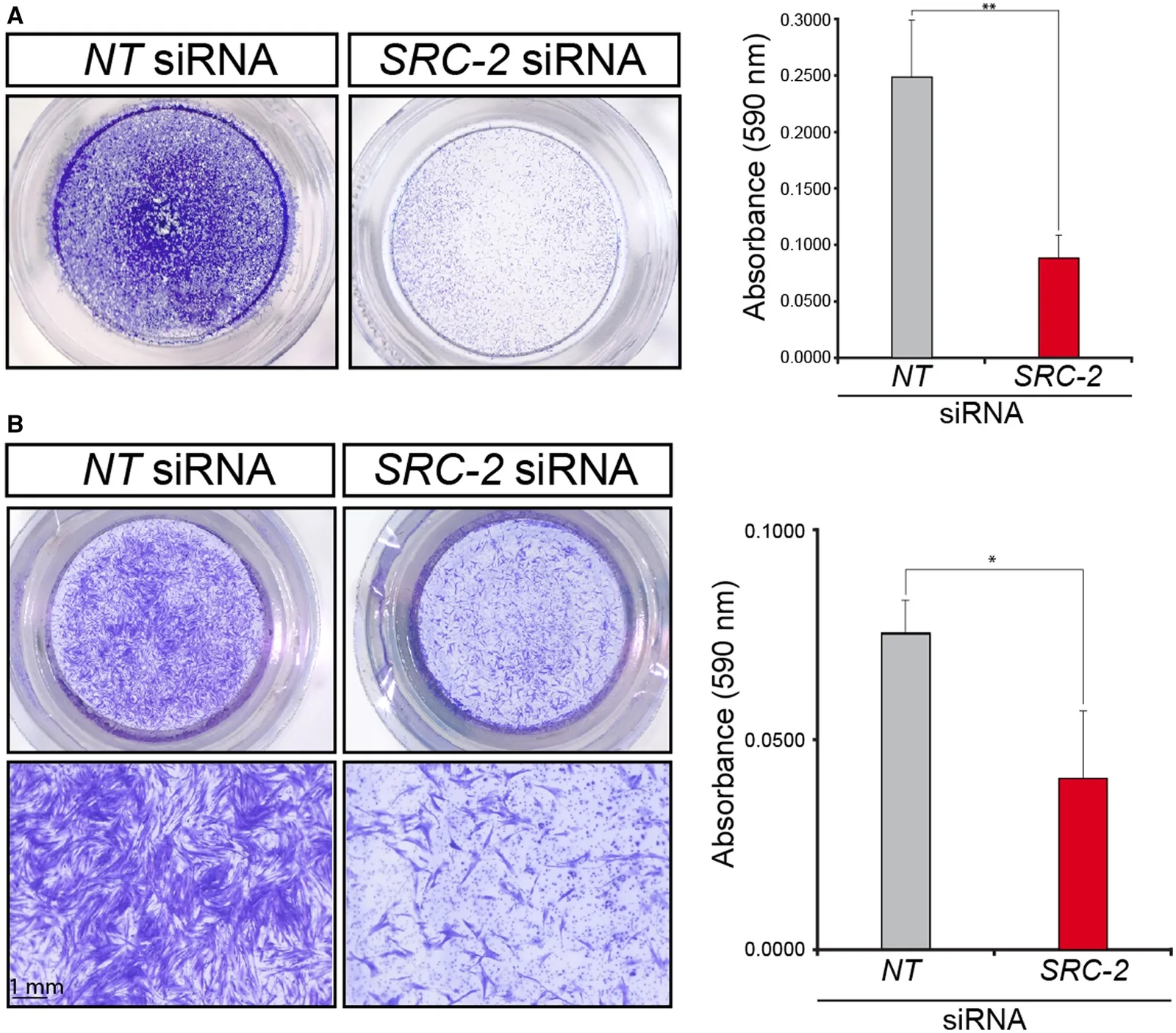
Reduction of steroid receptor coactivator-2 (SRC-2) levels significantly attenuates the migratory and invasive capacity of immortalized human endometriosis epithelial cells (iHEECs). (A) Left panels show a representative result of crystal violet staining from a transwell assay using iHEECs in which nontargeting (*NT*) or *SRC-2* small interfering RNAs (siRNAs) are transfected. Note the obvious reduction in crystal violet staining for *SRC-2* siRNA transfected iHEECs, indicating a significant reduction in the number of migrated iHEECs when SRC-2 levels are reduced. The histogram on the right represents the quantitation of the staining results in panel (A). Results are displayed as the mean of crystal violet absorbance (590 nm) for the iHEECs transfected with *NT* or *SRC-2* siRNAs. (B) Top left panels show a typical crystal violet staining result for the transwell invasion assay for iHEECs transfected with *NT* or *SRC-2* siRNAs. Note the significant decrease in iHEEC crystal violet staining following *SRC-2* siRNA transfection, indicative of decreased invasive capacity. The bottom panels represent higher power magnifications of subfields in the corresponding top panels. The histogram on the right represents the quantitation of the crystal violet stain eluted from invasive iHEECs previously transfected with *NT* or *SRC-2* siRNAs. The above results are shown as the mean ± standard deviation and are representative of three separate experiments; *p-value <0.01; and **p-value <0.001.

### The iHEEC transcriptome is significantly altered following SRC-2 knockdown

As SRC-2 is a transcriptional coactivator, genome-scale RNA profiling was performed to identify the downstream genes and signaling networks that are predicted to mediate SRC-2’s role in iHEEC pathogenicity. For the RNA-seq experimental design, iHEEC cells were cultured in six-well plates to 70% confluency before cells were transfected with *NT* or *SRC-2* siRNAs; triplicate wells for each of the two siRNA transfection groups were used. Forty-eight hours post-transfection, iHEECs were harvested for total RNA isolation and sequencing (Maurya et al., 2022). Differentially expressed genes between the two treatment groups are listed in the Excel spreadsheet in the supplementary section (Supplementary Folder 1). Importantly, the expression of the *SRC-2* gene is significantly downregulated (Log_2_ FC: −2.40) in the DEG list (yellow highlighted row).

The total number of DEGs that met the predetermined FDR (≤0.05) and FC (≥1.2) cutoffs was 1,725 (675 upregulated [39.1%] and 1,050 [60.9%] downregulated) (Figure 4; Supplementary Folder 1). Significantly, principal component analysis demonstrated a clear segregation of the *NT* and *SRC-2* siRNA-transfected iHEEC groups (Figure 4A), indicating that efficient *SRC-2* knockdown leads to marked changes in the iHEEC transcriptome. In the case of the volcano plot (Figure 4B), the following downregulated genes are annotated: actin alpha 2, smooth muscle (*ACTA2*); nuclear receptor coactivator-2 (*NCOA2*); *S100A10*; and leucine-rich repeat-containing G-protein coupled receptor 5 (*LRG5*); whereas the following upregulated genes are indicated: tissue factor pathway inhibitor 2 (*TFPI2*); interleukin 11 (*IL11*); sry-box transcription factor 17 (*Sox 17*); and paired box gene 8 (*PAX8*). These genes were highlighted to showcase the broad spectrum of potential biological functions that are significantly changed as a result of reducing *SRC-2* levels in iHEECs, many of which have been previously reported to be dysregulated in human endometriosis tissue (Chen et al., 2020; Dimitriadis et al., 2006; Fonseca et al., 2023; Yang et al., 2023; Zhu et al., 2026). Specific biological, cellular, and molecular pathway analysis of DEGs from comparing the iHEEC transcriptomes of the *NT* and *SRC-2* siRNA transfected groups revealed a significant enrichment of genes associated with actin and cytoskeletal organization in the downregulated DEG dataset (Figure 4C and D, and Figure S4). These results strongly suggest that SRC-2 is essential for these cellular processes, which are closely linked to the progression of endometriotic lesion adhesion, migration, invasion, and expansion in vivo (Albertsen & Ward, 2017; Arendt et al., 2025). To underscore the utility of this RNA-seq dataset in the context of actin cytoskeletal biology, we focused on S100A10 (Figure 4B), which is an essential regulator of actin cytoskeletal organization (Benaud et al., 2004; Jung et al., 2010; Liu et al., 2015; Sayeed et al., 2013).

**Figure 4:**
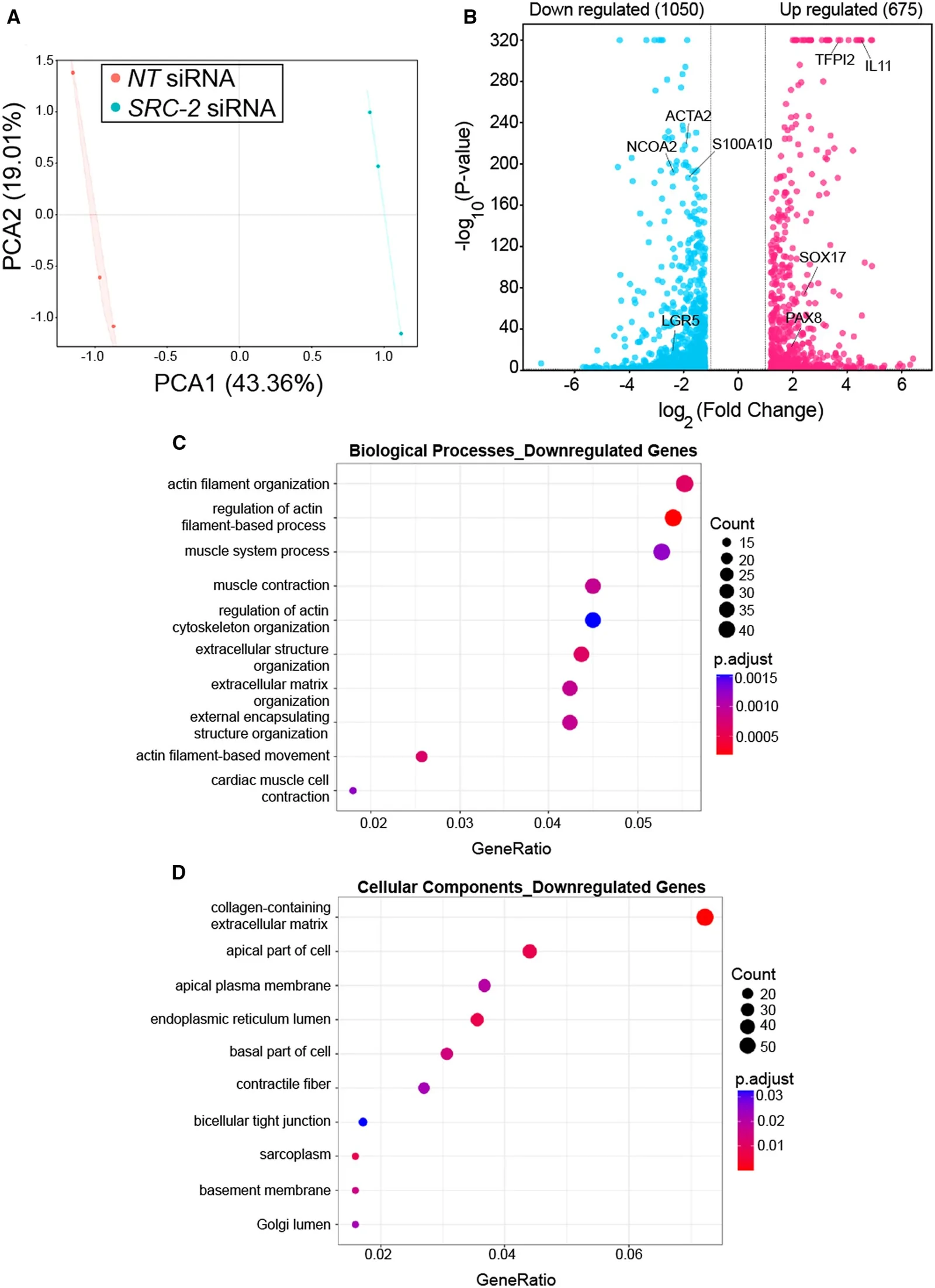
The human endometriotic epithelial transcriptome is significantly altered following steroid receptor coactivator-2 (*SRC-2*) knockdown. (A) Principal component analysis demonstrates a wide segregation of the triplicate nontargeting (*NT*) small interfering RNA (siRNA) and *SRC-2* siRNA-transfected immortalized human endometriosis epithelial cell (iHEEC) groups. (B) The volcano plot graphically displays the total number of genes differentially expressed between the *NT* siRNA and *SRC-2* siRNA treatment groups. Significance (*p*-value) versus fold-change are plotted on the y- and x-axis, respectively. The total number of genes differentially expressed between the two groups is 1,725 (1,050 downregulated and 675 upregulated). In the downregulated group the following genes are indicated: *ACTA2*, *NCOA2*, *S100A10*, and *LRG5*; in the upregulated the following genes are indicated: *TFPI2*, *IL11*, *SOX17*, and *PAX8*. (C) Dot-plot of downregulated genes that are enriched within the differentially expressed gene (DEG) dataset and stratified according to biological processes (BP). Note that the expression of genes involved in cytoskeletal organization is significantly altered following *SRC-2* knockdown in iHEECs; the predominance of biological functions associated with actin filament and cytoskeleton organization is significant. (D) Using the same downregulated gene dataset from the DEG, the dot-plot for the cellular component (CC) module reveals an overrepresentation of genes linked with extracellular matrix and apical cellular changes.

### S100A10 protein expression is predominant in epithelial cells of endometriotic lesions and is responsive to SRC-2

Immunohistochemical analysis shows that S100A10 is exclusively expressed in the epithelial cellular compartment of baboon endometriotic lesions (Figure 5A–C). Significant S100A10 expression was not detected in matched eutopic endometrium except for low levels of expression in blood vessels (Figure 5D–F) (Chehab et al., 2017; Holthenrich & Gerke, 2018; Luo et al., 2017). As SRC-2 is expressed in the epithelium of the eutopic endometrium (Figure 1), these results suggest SRC-2 controls different transcriptional programs in the epithelium of the eutopic and ectopic endometrial tissue. A similar S100A10 expression pattern is observed in experimentally induced mouse endometriotic lesion (Figure S5). Of note, quantitative PCR demonstrates that *SRC-2* knockdown in iHEECs results in a significant reduction in the *S100A10* levels (Figure 5G), which is also confirmed at the protein level by western blot analysis (Figure 5H). These results are further confirmed by immunocytochemistry which shows a marked reduction in S100A10 protein expression in iHEECs following siRNA-mediated *SRC-2* knockdown (Figure 5I). Similar to SRC-2, *S100A10* knockdown in iHEECs significantly attenuates the sustained viability as well as the clonogenic, migratory, and invasive properties of this endometriotic epithelial cell type (Figure 6), suggesting that this SRC-2 responsive gene is critical for many of pathogenic cellular behaviors associated with the endometriotic epithelial cell.

**Figure 5:**
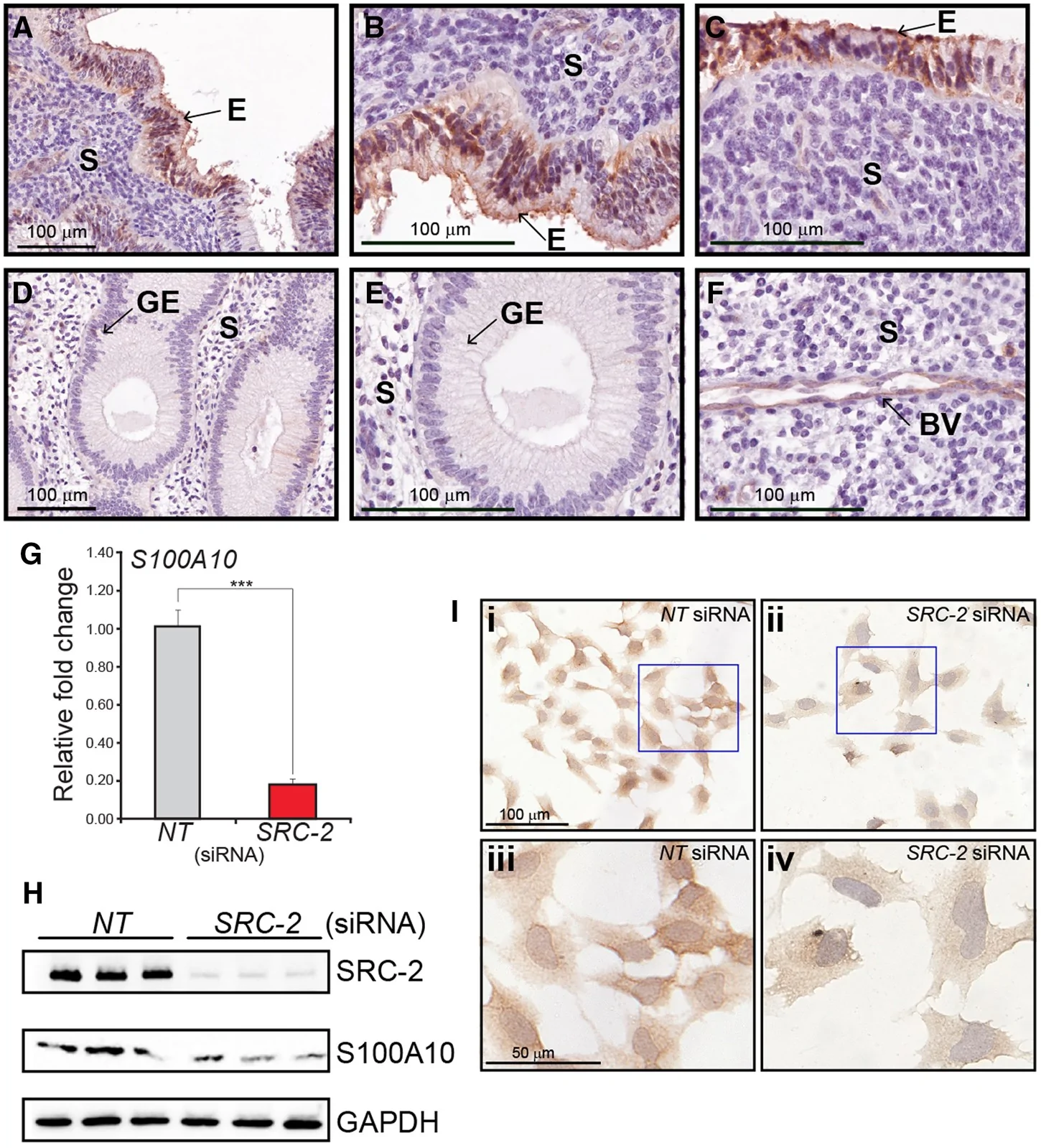
S100A10 is predominantly expressed in endometriotic epithelial cells and is dependent on steroid receptor coactivator-2 (SRC-2) expression. (A–C) Increased magnification of baboon endometriotic lesion tissue that is immunohistochemically stained for S100A10 expression. Note the expression of S100A10 is localized to the epithelial (E) cell layer of the endometriotic lesion; S100A10 is not expressed in the underlying stromal (S) cell layer. (D–F) Increased magnification of baboon eutopic endometrial tissue immunohistochemically stained for S100A10 expression. Note the absence of S100A10 expression in the stromal and glandular epithelial compartments of eutopic endometrial tissue. (F) A low level of S100A10 expression is detected within the blood vessel (BV) wall. The immunohistological results represent a replicate from four replicates analyzed. (G) Quantitative real-time PCR analysis shows that *SRC-2* knockdown results in a significant reduction in the *S100A10* levels in immortalized human endometriosis epithelial cells (iHEECs). The results are presented as the mean ± standard deviation and are representative of three separate experiments; ***p-value <0.0001. (H) Western immunoblot confirms that *SRC-2* knockdown results in a significant decrease in S100A10 protein expression; GAPDH serves as a loading control. Three samples per nontargeting (*NT*) and *SRC-2* small interfering RNA (siRNA)-treated group are shown; results represent one experiment of a triplicate. (I) Immunocytological staining confirms that S100A10 protein expression is significantly responsive to SRC-2 levels; (i) shows clear S100A10 immunopositivity in iHEECs following a 48-hr transfection period using *NT* (control) siRNAs; (ii) shows a significant reduction in S100A10 immunopositivity in iHEECs following a 48-hr transfection period using *SRC-2* siRNAs; (iii) higher power magnification of a subfield demarcated by the blue square in panel (i). Note the marked expression of S100A10 protein in the cytoplasm. (iv) Higher power magnification of the subfield delineated by the blue square in panel (ii). Note the diminished immunoreactivity for S100A10 protein in iHEECs transfected with *SRC-2* siRNAs. Scale bar in panel (i) applies to panel (ii), whereas that in panel (iii) applies to panel (iv).

**Figure 6:**
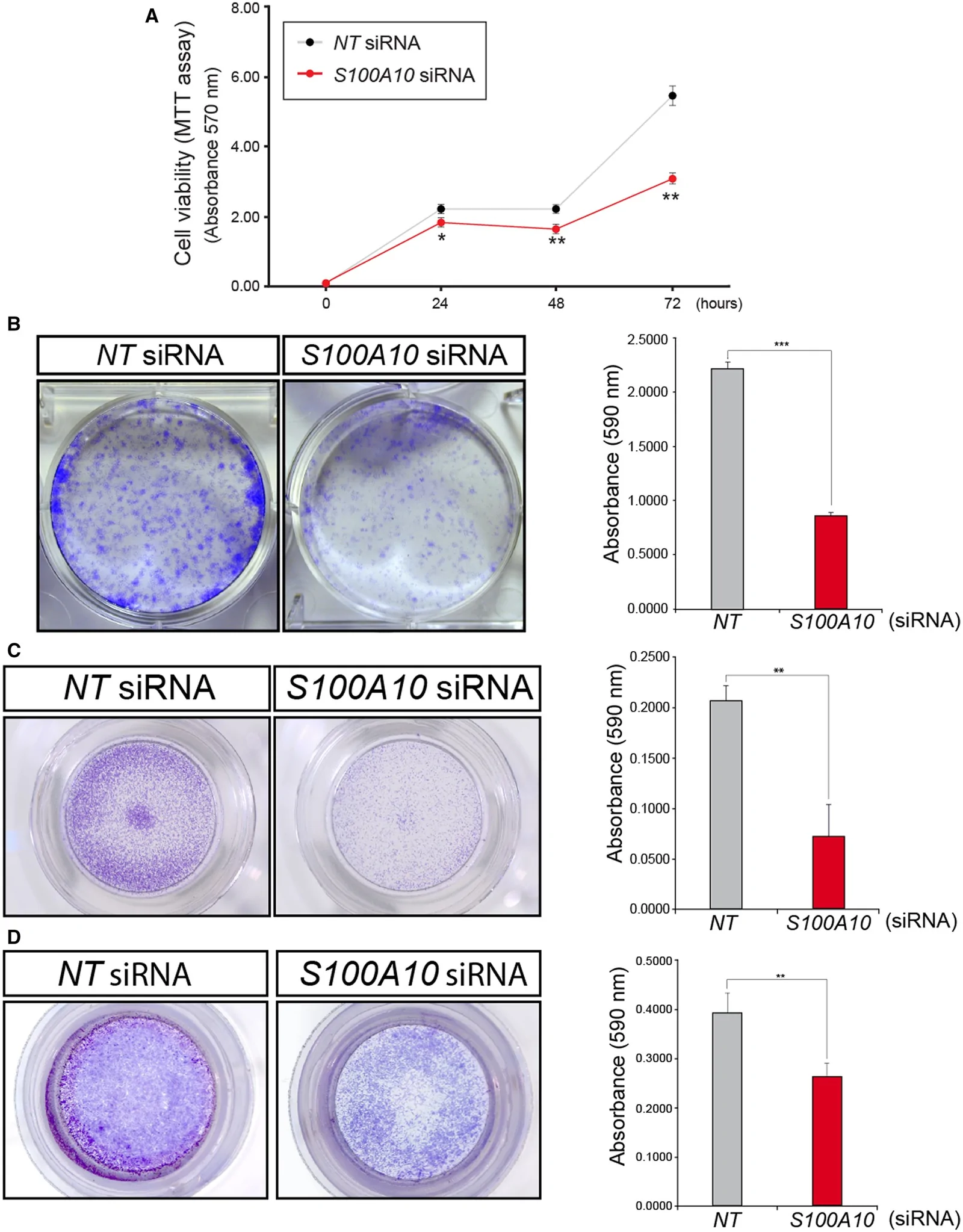
Pathogenetic properties of immortalized human endometriosis epithelial cells (iHEECs) are compromised following *S100A10* knockdown. (A) The MTT (3-[4,5-dimethylthiazol-2-yl]-2,5-diphenyltetrazolium bromide) assay shows that sustained viability is reduced in iHEECs following *S100A10* knockdown. (B) The colony-formation assay (left panels) shows a marked decrease in crystal violet staining for iHEECs with reduced *S100A10* levels. Histogram (right) quantitates the difference in colony formation ability through measure of mean absorbance (590 nm) of the crystal violet containing eluent from colonies of iHEECs transfected with nontargeting (*NT*) or *S100A10* small interfering RNAs (siRNAs). (C) Transwell migration assay (left panels) shows that full migratory ability of iHEECs depends on normal S100A10 levels. Corresponding histogram (right) displays the quantitation of the transwell migration assay result, which shows the mean absorbance (590 nm) for the eluent from migratory iHEECs previously transfected with *NT* and *S100A10* siRNAs. (D) A typical transwell invasion assay result (left panels) shows a marked decrease in crystal violet staining for iHEECs transfected with *S100A10* siRNAs. Histogram (right) quantitatively confirms the staining result in which the mean absorbance (590 nm) for eluent obtained from iHEECs transfected with *S100A10* siRNAs is significantly reduced compared with iHEECs transfected with *NT* siRNAs. The results shown in the above histograms represent the mean ± standard deviation and are representative of three separate experiments; *p-value <0.01; **p-value <0.001; and ***p-value <0.0001.

## Discussion

The described studies show that the predominant cell-type that expresses SRC-2 protein in the primate and murine endometriotic lesion is the epithelial cell. Using the established iHEEC line (Bono et al., 2012), we reveal that SRC-2 is indispensable for many of the pathogenetic properties of endometriotic epithelial cells, including sustained viability, proliferation, migration, and invasion. These results are in line with past reports that describe similar functions for SRC-2 in other invasive cell types, from transformed epithelial cells to embryonic-derived epithelial trophoblasts (Kishimoto et al., 2005; Maurya et al., 2025; Meng et al., 2021). However, it should be noted that only one ectopic endometriosis epithelial cell line (iHEEC) modeling one type of ectopic endometriosis (ovarian endometrioma) was used in these studies. Therefore, the cell-based investigations described here will be further strengthened and expanded upon in the future by using additional epithelial cell lines that model the other endometriosis subtypes, such as superficial peritoneal endometriosis.

Genome-wide transcriptomics in conjunction with siRNA-mediated silencing show that the endometriotic epithelial cell significantly relies on SRC-2 for the maintenance of its transcriptome, the transcriptional underpinning of its pathogenic cellular behaviors. In particular, gene expression programs linked with actin cytoskeleton organization are significantly altered in iHEECs in response to SRC-2 depletion. This observation is important in the context of endometriotic lesion expansion and invasion as actin cytoskeleton reorganization in the ectopic endometriotic cell is crucial for its focal adhesion properties, mechanosensing abilities, membrane trafficking capabilities, extracellular matrix invasion behaviors, motility, epithelial-to-mesenchymal transition, and cytokinesis at distant anatomic locations, such as the pelvic peritoneal wall (Albertsen & Ward, 2017; Arendt et al., 2025).

To demonstrate the utility of this transcriptomic dataset in linking new signaling mechanisms with SRC-2 action in the ectopic endometriotic epithelial cell, we focused on S100A10 (also termed p11, cellular ligand of annexin II, and calpactin I light chain) (Okura et al., 2023; Rescher & Gerke, 2008). A small 97 amino acid protein, S100A10 comprises a heterotetramer containing two light chain S100A10 proteins complexed with two heavy chain annexin A2 proteins (Kwon et al., 2005; Menke et al., 2004). Expressed in the cytoplasm and/or nucleus, S100A10 is an integral part of the cellular scaffolding that interacts with plasma membrane proteins as well as regulating the cellular actin architecture (Jung et al., 2010; Sayeed et al., 2013). In complex with annexin A2, S100A10 is involved in both intracellular and extracellular signaling processes through trafficking of proteins to the plasma membrane. Also acting as a cell surface receptor itself (Seo & Svenningsson, 2020), S100A10 can serve as a critical plasminogen receptor for plasminogen and its activators (tPA and uPA) (Madureira et al., 2016; Miller et al., 2017; Phipps et al., 2011), which together generate plasmin, a broad-spectrum serine protease that is critical for fibrinolysis, extracellular matrix remodeling, and tissue invasion (Surette et al., 2011). These are the very biological processes that are used for endometriotic lesion establishment and expansion (Ellis & Wood, 2026). Because S100A10 overexpression is reported for numerous cancers (Bharadwaj et al., 2021; Hedhli et al., 2012; Madureira et al., 2012; Noye et al., 2018; Saiki & Horii, 2019; Yanagi et al., 2020), this protein has been promoted by many groups as an independent biomarker across multiple tumor types (Bydoun et al., 2018; Christensen et al., 2019; Domoto et al., 2007; Gao et al., 2022; Huang & Tu, 2024; Lokman et al., 2016; Ma et al., 2022; Shang et al., 2013; Tantyo et al., 2019). Similarly, we found that *S100A10* levels are significantly elevated in previously reported expression profiling datasets derived from human ectopic endometriotic lesions (Figure S6) (Eyster et al., 2007; Hever et al., 2007), suggesting that S100A10 has a role in endometriosis progression. Our cell-based studies support this supposition as S100A10 is essential for optimum iHEEC sustained viability, migration, and invasion—all of which are dependent on normal levels of S100A10, which in turn is controlled by SRC-2.

For the studies described here, S100A10 is representative of downregulated genes classified within the actin cytoskeleton organization group; however, further examination of the transcriptomic dataset reveals that the expression of genes linked with other important biological functions are also markedly changed with *SRC-2* knockdown. Although beyond the scope of further investigation here, these include genes associated with Wnt signaling (i.e., the Wnt ligands: *WNT6*, *WNT2*, and *WNT10B*; secreted frizzled-related protein 4 [*SFRP4*; a Wnt antagonist]; Dickkopf-2 [*DKK2*; a *LRP5/6* antagonist]; Leucine-rich repeat-containing G-coupled receptor 5 [*LRP5*; a Wnt coreceptor, intestinal/epithelial stem cell marker]; frizzled-related protein [*FRZB*; a Wnt inhibitor]; and frizzled-related receptor 4 [*FZD4*; a Wnt signal transducer]). All components of the Wnt pathway detected in this dataset are uniformly downregulated, representing an eight-gene cluster of Wnt ligands, antagonists, and receptors. The simultaneous downregulation of both Wnt ligands and Wnt pathway antagonists may initially appear contradictory. However, this pattern reflects global Wnt pathway transcriptional dependency on SRC-2 coactivation. Downregulation of LGR5 is particularly notable, suggesting that reduced epithelial stem cell–like properties in endometriotic cells upon SRC-2 depletion may occur. For upregulated genes in the DEG dataset, a prominent enriched biology is proinflammatory cytokine and chemokine induction, which include *IL6* (IFCI: +4.47), *IL11* (IFCI: +4.53), *CSF3* (IFCI: +4.89), CSF2 (GM-CSF, IFCI: +3.53), *CXCL8* (IL8, IFCI: +3.35), and *CCL3* (IFCI: +6.32). This cytokine profile bears resemblance to a Senescence-Associated Secretory Phenotype (SASP) (Acosta et al., 2008; Birch & Gil, 2020; Childs et al., 2017), which encompasses *IL6, IL8*/*CXCL8*, *GM-CSF2*, and multiple chemokines. The fact that *SRC-2* knockdown is predicted to trigger this inflammatory program suggests that SRC-2 normally functions as a transcriptional suppressor of inflammatory cytokine expression in the endometriotic epithelium.

In summary, the present investigation strongly establishes the SRC-2 coactivator—and by extension its responsive transcriptome—as a driver of many pathogenic epithelial cellular processes ascribed to the expansion of ectopic endometriotic lesions. These results do not, however, discount the contribution of stromal-derived SRC-2 to ectopic endometriosis pathology—a subject for future investigation. In addition, future study will make use of conditional knockout mouse models to confirm in vivo the importance of SRC-2, S100A10, and/or other SRC-2-responsive molecular targets in ectopic endometriotic lesion establishment and expansion.

## Supplementary Material

xaag125_Supplementary_Data

## Data Availability

The data underlying this article are available in the article and in its online supplementary materials. The sequencing data have been deposited in GEO under accession code GSE334431.
